# 

**Published:** 1921-01-15

**Authors:** 


### Matrons' and Sisters' Appointments.

KENT AND CANTERBURY HOSPITAL.—Miss Annie F. Purchas has been appointed matron. She was trained at the Royal Devon and Exeter Hospital, and for three years was matron of No. 4 War Hospital, Exeter, afterwards becoming matron of Clopton War Hospital, Stratford-on-Avon. She has been assistant matron of the Kent and Canterbury Hospital since 1920. Miss V. Blake has been appointed night sister and Miss F. Roberts sister of the medical and eye wards. Both were trained at the Royal Devon and Exeter Hospital.

BIRMINGHAM ROYAL INSTITUTION FOR THE BLIND.—Miss Jean S. Miller has been appointed sister. She was trained in general nursing at the Royal Infirmary, Liverpool, in mental nursing at the County Asylum, Chester, and for four years studied eye nursing at the Eye Hospital, Shrewsbury. She has since been senior sister to the Ear and Throat Hospital, Birmingham, theatre and surgical sister in Serbia and Greece, medical sister in France, and sister-in-charge of typhus patients in Poland.

STOKE-ON-TRENT UNION INFIRMARY.—Miss E. H. Churchman, Miss R. Spry, and Miss C. S. Allchin have been appointed sisters. Miss Churchman was trained at Erdington Infirmary, Birmingham, where she was later temporary sister; Miss Spry was trained at Hackney Union Infirmary and the Rotunda Hopital, Dublin, and has been staff nurse Q.A.I.M.N.S.R.; and Miss Allchin was trained at Chesterfield Royal Infirmary and the Jessop Hospital, Sheffield, at which latter institution she was subsequently staff nurse. All three have the certificate of the Central Midwives Board.

MATERNITY AND CHILDREN'S HOSPITAL, SPRINGFIELD, ROCHDALE.—Miss Ellen Hollinshead has been appointed sister. She was trained at the Burton-on-Trent General Infirmary and Dispensary, and has subsequently been sister at the Royal Portsmouth Hospital, sister at the Milton Hospital, Portsmouth, and health visitor at Middleton and at Mansfield-Woodhouse under the Nottinghamshire Urban District Council.

### Personalia.

SIR EDWARD NAPIER BURNETT, K.B.E., Director of Hospital Services, British Red Cross Society, has been appointed a Knight of Grace of the Order of St. John of Jerusalem.

MISS DORA M. SIMMONDS has been appointed almoner to the Royal Victoria and West Hants Hospital, Bournemouth. Miss Simmonds was assistant almoner to St. Mary's Hospital, Paddington, for two years. She left this post to become Deputy Principal Women's Royal Naval Service, and subsequently was third almoner to the Royal Free Hospital. She was trained by the Hospital Almoners' Council.

THE following appointments have been made at the Hampstead General and North-West London Hospital: House physician, Mr. Philip Hudson, M.B., B.S.Lond., M.R.C.S., L.R.C.P.; house surgeon, Mr. Roland H. Fulton, M.B., Ch.B., New Zealand; casualty medical officer, Mr. G. S. Lawrence, M.A., M.B., Ch.B. Aberdeen; casualty surgical officer, Mr. W. A. Miller, D.S.O., M.C., M.B., Ch.B. (Edin.).

A COMMITTEE, consisting of the Master of the Rolls, Ireland, Sir Edward Coey Bigger, Mr. Stephen Grehan, Sir James Johnston, Mr. T. B. Lillis, Sir Henry McLaughlin, Mrs. McMordie, and Mr. T. Laurence Waldron, has been appointed by the Chief Secretary for Ireland to advise on the distribution of the National Relief Fund grant towards the war deficits of voluntary hospitals in Ireland.

AT the Nosocomia Lodge of Freemasons on January 7, Mr. A. H. Coughtrey, known in hospital life as the librarian at St. Bartholomew's, was installed as Master, succeeding Mr. G. A. Arnaudin in the chair. The Lodge is for the convenience of hospital workers, and several names in the list of officers for 1921 will be recognised by our readers. Mr. J. Courtney Buchanan, C.B.E., is the new Senior Warden, and Mr. W. G. Garner the Junior Warden. Messrs. E. A. Attwood and Stanley Smith continue respectively as Treasurer and Secretary. Mr. W. J. Chichele Nourse, F.R.C.S., is Director of Ceremonies, and the other senior offices are filled by Messrs. W. M. Cooper, George Watts, H. W. Burleigh, G. E. Pitt, J. A. Hamlyn, E. Williamson, and Ian Mackay. At the banquet following the meeting there were several notable guests, including Sir D'Arcy Power, K.B.E., Mr. Girling Ball, F.R.C.S., Dr. Spilsbury, and representatives of the Æsculapius, Rahere, Cheselden, Dacre, and other lodges.

